# Lysine120 Interactions with p53 Response Elements can Allosterically Direct p53 Organization

**DOI:** 10.1371/journal.pcbi.1000878

**Published:** 2010-08-05

**Authors:** Yongping Pan, Ruth Nussinov

**Affiliations:** 1 Basic Science Program, Science Applications International Corporation-Frederick, Inc., Center for Cancer Research Nanobiology Program, National Cancer Institute-Frederick, Frederick, Maryland, United States of America; 2 Sackler Institute of Molecular Medicine, Department of Human Genetics and Molecular Medicine, Sackler School of Medicine, Tel Aviv University, Tel Aviv, Israel; Sabanci University, Turkey

## Abstract

p53 can serve as a paradigm in studies aiming to figure out how allosteric perturbations in transcription factors (TFs) triggered by small changes in DNA response element (RE) sequences, can spell selectivity in co-factor recruitment. p53-REs are 20-base pair (bp) DNA segments specifying diverse functions. They may be located near the transcription start sites or thousands of bps away in the genome. Their number has been estimated to be in the thousands, and they all share a common motif. *A key question is then how does the p53 protein recognize a particular p53-RE sequence among all the similar ones*? Here, representative p53-REs regulating diverse functions including cell cycle arrest, DNA repair, and apoptosis were simulated in explicit solvent. Among the major interactions between p53 and its REs involving Lys120, Arg280 and Arg248, the bps interacting with Lys120 vary while the interacting partners of other residues are less so. We observe that each p53-RE quarter site sequence has a unique pattern of interactions with p53 Lys120. The allosteric, DNA sequence-induced conformational and dynamic changes of the altered Lys120 interactions are amplified by the perturbation of other p53-DNA interactions. The combined subtle RE sequence-specific allosteric effects propagate in the p53 and in the DNA. The resulting amplified allosteric effects far away are reflected in changes in the overall p53 organization and in the p53 surface topology and residue fluctuations which play key roles in selective co-factor recruitment. As such, these observations suggest how similar p53-RE sequences can spell the preferred co-factor binding, which is the key to the selective gene transactivation and consequently different functional effects.

## Author Summary

p53-response elements (p53-REs) are 20 base pairs (bps) DNA segments recognized by the p53 transcription factor (TF). They are found in promoters and enhancers across the genome and are associated with genes that have diverse functions. Because the DNA sequences of p53-REs can be very similar to each other, differing by as little as one or two bps, it is challenging to understand how p53 distinguishes between these to activate a specific function. Here we show that even a slight RE sequence change can be sufficient to elicit allosteric structural and dynamic perturbations in the p53 which propagate to other binding sites, and as such are expected to affect co-regulator recruitment. Among the major interactions between p53 and its REs involving Lys120, Arg280, and Arg248, the Lys120 interaction partners vary less than interactions between other residues. The outcome of our simulations of six p53-RE complexes shows that the variance of the interaction patterns triggers changes in the organization of tetrameric p53 and of residues away from the interaction sites. Subsequent events can depend on the level and post-translational states of co-regulators that are able to bind the unique p53 surface caused by the specific p53-RE binding.

## Introduction

p53-response elements (p53-REs) are two 10-bp palindromic DNA segments with the consensus sequence of 5′-Pu1Pu2Pu3C4(A/T)5(A/T)5′G4′Py3′Py2′Py1′-3′ for each of the two half sites, where Pu and Py stand for purine and pyrimidine bases, respectively [1], [2]. The two half sites can be separated by as many as 20 bps [1]–[6]. Hundreds of p53-REs have been identified [2], [5], and the numbers continue to grow [7]. Many of these are known to be related to regulation of genes involved in cellular pathways such as apoptosis, cell cycle arrest and senescence [8], [9]. However, upon stimulation only a small subset are selectively activated for transcriptional activation or repression through sequence-specific binding to tumor suppressor p53. Understanding the factors that determine the selective activation is crucial for deciphering the complex gene regulation by p53 [7], [10]–[14]. Binding affinities of functionally-diverse p53-REs showed that apoptosis-related p53-REs have higher affinities than cell cycle arrest-related p53-REs; however, at the same time, the affinities do not always correlate with functional effects [7], [12], [15], [16]. Spacer sizes also affect affinities: in spacers consisting of three or more bps, the two 10-bp half-sites are on opposite faces of the DNA [17], suggesting specific p53-RE interactions only with a single half-site, which results in lower affinity [7], [17]. Although several structures are available [9], [18]–[23], they involve a few engineered p53-REs and do not explain the *in vivo* selectivity. *In vivo*, p53-RE binding is affected by chromatin packaging epigenetic events known to be a key factor in RE occupancy [24], [25]. Nonetheless, even assuming genomic p53-REs availability, the question of the selective recognition by p53 still remains [12], [13].

Allostery is key to cellular signal transduction [26]–[30]. Mechanistically [12], [13], allostery can play a role either via protein co-factors binding to p53 prior to RE binding as could be in HIF-1 regulation of p53 and p300 [31], or ASPP family binding [32]; or via allostery-induced by RE sequences [33]–[37], or spacer sizes as in the pituitary-specific POU domain factor Pit-1 [38], in both cases through preferential interactions with certain side chain conformations [34]. In p53, RE bp changes were observed to relate to transactivation [39]. In the glucocorticoid receptor (GR) [40], [41], single bp changes were shown to allosterically affect GR conformational changes. These were amplified by ligand binding and propagated to the co-regulator binding site. Allosteric effects can shift the population toward co-factor binding-favored states. DNA methylation can lead to packing of the genome, making the REs unavailable; but it was also proposed to change the affinities of the REs [42], [43] either via direct interactions, or through allosteric effects on the DNA or the protein. In proteins, covalent modifications such as phosphorylation, glycosylation, and acetylation are well established to be allosteric effectors.

The tetrameric p53 DNA-binding domains (DBD) are responsible for specific RE binding. However, the impact of the DNA sequence on the binding patterns, specificities and complex conformation has been studied only for the central 4 bps [44], [45]. Computational studies revealed that variation of the central four bps in the half site which contained the C(A/T)(T/A)G, conserved in most REs, resulted in conformational changes in the DNA and the DBD [45]. However, the impact of RE sequence variation in other bps on the complex organization and its dynamic properties is largely unknown due to the sparseness of available crystal structures. Here, using molecular dynamics (MD) simulations we study the conformational and dynamic consequences of p53 binding to six diverse p53-REs. We focus on the impact of specific interactions of Lys120, Arg280 and Arg248 with DNA as these are the most crucial for binding. We find that p53 Lys120-DNA interactions can change dramatically depending on the bp at positions 1-3 of the quarter site, which in turn affects the Arg280 binding. We find that such binding pattern changes at the DNA-protein interface have allosteric effects in terms of the p53 tetrameric organization and the fluctuations of residues on the p53 surface away from the DNA binding site. We propose that this combined allosteric effect could hold the key to selective transcriptional activation by the degenerate p53-REs and can serve as a paradigm for selective activation of transcription factors [13].

## Results

**Figure 1:**
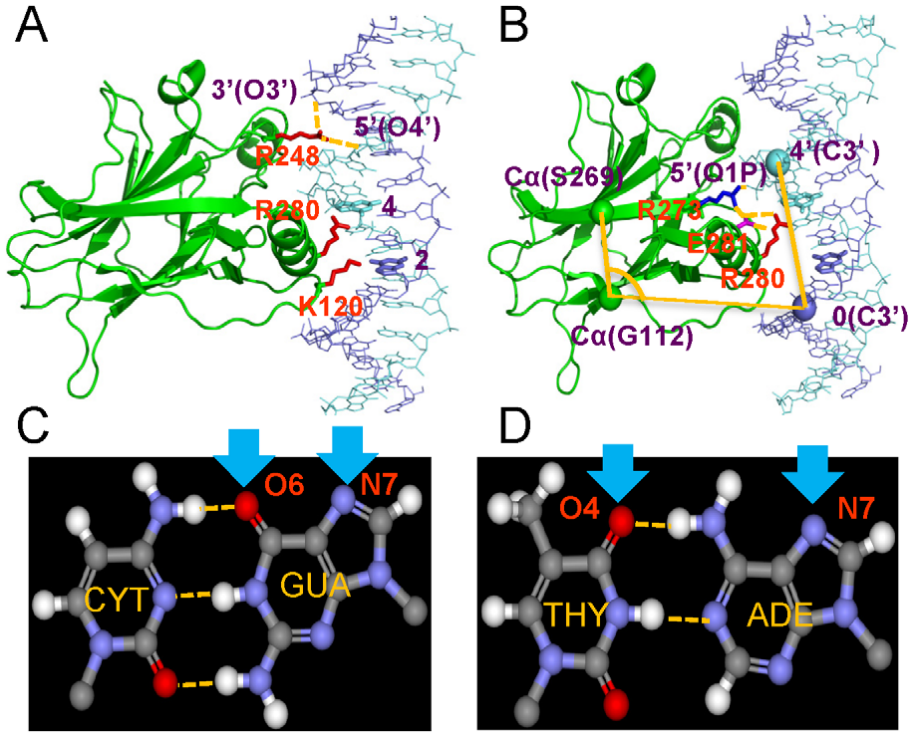
Illustration of the monitored p53 core domain-REs specific interactions and p53 intra-domain interactions. The DNA quarter-site bases are labeled as Pu1Pu2Pu3C4(A/T)5 and as Y1′Y2′Y3′G4′(T/A)5′ for the complementary chain. (A) Lys120 and Arg280 interact with the bases from the major groove while Arg248 interacts from the minor groove. Lys120 can potentially interact with bases at base positions 1–3 in a quarter site. The G bases that formed hydrogen bond with Lys120 and Arg280 are shown in thick sticks. Depending on the base identity, Lys120 may form a three-centered hydrogen bond with a G base (C) or a two-centered hydrogen bond with either a T or A base (D). Arg280 normally interacts with the G base at the 4′^th^ position in a quarter site that is largely conserved. Two monitored distances for Arg248 interaction with the DNA backbone are shown. (B) The salt bridge network among the base, residues Arg280, Glu281, R273 and the DNA backbone in the crystal structures is shown in dashed lines. The angle that is monitored is defined as between atoms Cα of S269, Cα of G112 and C3′ of the nucleotide at position 0 of the respective quarter site. The dihedral angle is defined by the above three atoms plus the C3′ atom at the 4′ position of the DNA. The two protein atoms are located at the centers of the well structured β-sheets and the two DNA atoms are close to the quarter site that interacted with the corresponding p53 core domain. These atoms are shown in spheres. These geometrical parameters are expected to reflect the organizational changes of p53 with respect to DNA. (C) and (D) Hydrogen bonding pattern differences between base pairs AT and GC. Hydrogen bonding donors from the DNA bases are labeled. The arrows point to the coming direction of the Lys120 or Arg280 residues from the p53.

Six naturally-occurring p53-REs were selected, two each from the cell cycle arrest, DNA repair and apoptosis functional groups (Table 1). These REs differ from the consensus sequences by 1–3 bps (Table 1). To analyze the impact of the sequences on p53 binding, conformations and organization, hydrogen bond (HB) distances for p53 residues Lys120, Arg280, Arg248 and Arg273, DNA conformational differences, residue deviation and fluctuations in each quarter site (denoted as Q1, Q2, Q3 and Q4) and overall complex organizations were monitored. In the crystal structure Lys120 and Arg280 form HB with DNA bases in the major groove, while Arg248 anchors in the minor groove through electrostatic interactions (Fig 1a). The salt bridge network among Arg280, Glu281, and Arg273 (interacting with the DNA backbone) enhances the specific protein-DNA interactions (Fig 1b).

**Table 1:** Lys120 hydrogen bond percentage calculated from the last 20 ns of the trajectories.

	Q1					Q2					Q3					Q4				
	1	2	3	4	5	5′	4′	3′	2′	1′	1	2	3	4	5	5′	4′	3′	2′	1′
14-3-3σ	**A**	**G**	**G**	**C**	**A**	**T**	**G**	**T**	**g**	**C**	**c**	**A**	**c**	**C**	**A**	**T**	**G**	**C**	**C**	**C**
(cell cycle arrest)	12	87	30					0	15	91	0	3	0					81	73	0
GADD45	**G**	**A**	**A**	**C**	**A**	**T**	**G**	**T**	**C**	**T**	**A**	**A**	**G**	**C**	**A**	**T**	**G**	**C**	**T**	**g**
(DNA repair)	51	14	0					3	46	0	0	1	0					14	48	0
Noxa	**A**	**G**	**G**	**C**	**T**	**T**	**G**	**C**	**C**	**C**	**c**	**G**	**G**	**C**	**A**	**A**	**G**	**T**	**T**	**g**
(Apoptosis)	0	73	62					0	69	74	0	73	86					0	0	0
P21-5	**G**	**A**	**A**	**C**	**A**	**T**	**G**	**C**	**C**	**C**	**c**	**A**	**A**	**C**	**A**	**T**	**G**	**T**	**T**	**g**
(cell cycle arrest)	0	49	0					0	90	75	0	44	0					0	0	0
P53R2	**t**	**G**	**A**	**C**	**A**	**T**	**G**	**C**	**C**	**C**	**A**	**G**	**G**	**C**	**A**	**T**	**G**	**T**	**C**	**T**
DNA repair)	0	0	0					88	87	0	0	87	91					0	54	48
Puma	**c**	**t**	**G**	**C**	**A**	**A**	**G**	**T**	**C**	**C**	**t**	**G**	**A**	**C**	**T**	**T**	**G**	**T**	**C**	**C**
(Apoptosis)	0	0	0					0	85	0	0	33	0					0	59	1

A distance cutoff of 3.5 Å between the donor and acceptor heavy atoms was used in defining the hydrogen bond. Lower case letters indicate the base identity deviation from the consensus sequence.

### The specificity of Lys120 interaction with DNA is sequence-dependent

Lys120 can interact with bps at three positions (positions 1–3 in a quarter site) (Fig 1a). However, the interaction patterns can vary, depending on the base identity. With a G base, Lys120 can make three center HBs (Fig 1c). For C, Lys120 can make the same interactions with the G on the other chain, but the protein has to adjust its relative position. For an A or T, Lys120 can only make one HB with either base but not both because the two HB acceptors are 6–7 Å apart in a Watson-Crick bp (Fig 1d). The methyl group next to the T O4 atom can also influence the interactions.

**Figure 2:**
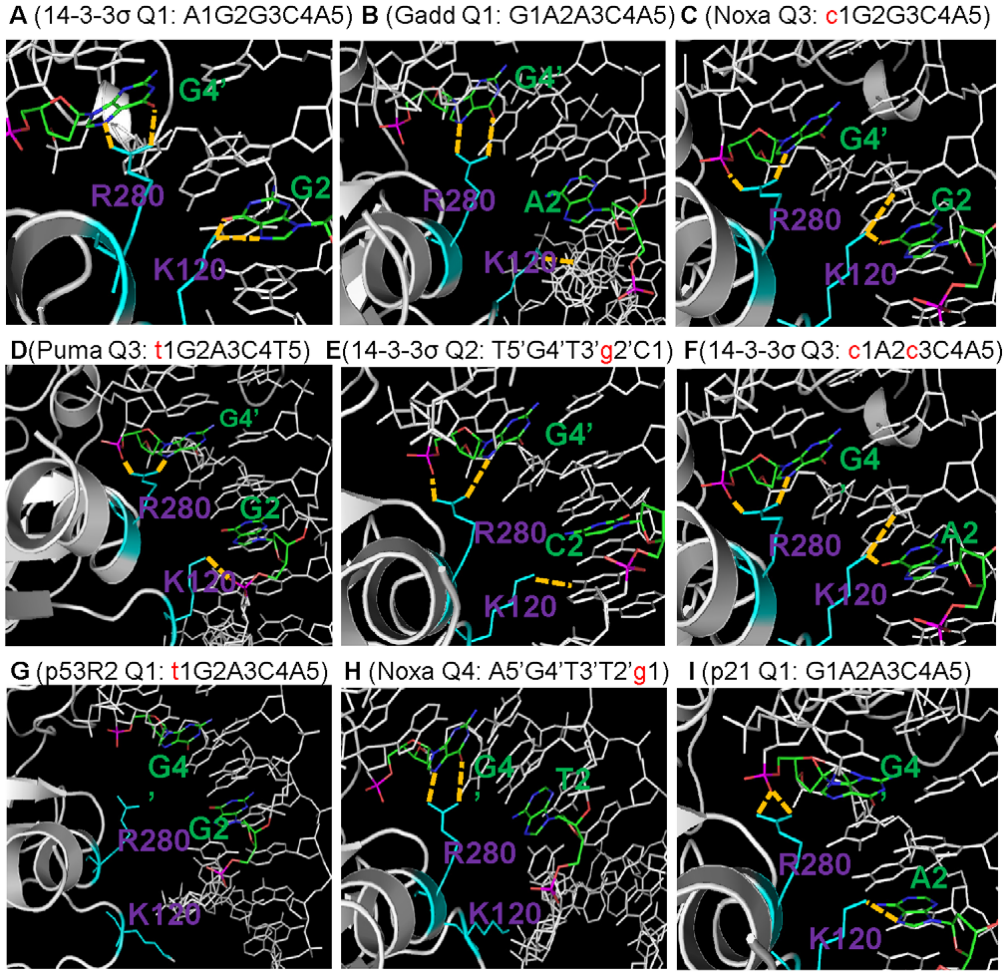
Average structures of the p53-DNA complex over the last 5 ns of the Lys120 and Arg280 binding sites. Lys120 and Arg280 are colored in cyan and the 2^nd^ and 4′^th^ bases are colored based on atom type. Hydrogen bonds formed between Lys120 and the 2^nd^ base or between Arg280 and the 4′^th^ base are shown in dotted yellow lines. The RE and its sequence for each selected structure are also listed on top of each panel. The calculations were performed with the CHARMm analysis module COOR DYNAMICS.

All six potential HB distances for the three bps were monitored (Fig. S1) and the percentage of distances less than 3.5 Å are summarized in Table 1. Fig 2 highlights the average local conformation of Lys120 and Arg280 for selected binding sites. The results show that (a) with a quarter site whose sequence conforms to the consensus, Lys120 interacted mainly with the central G or A base, as in the crystal structures (Table 1: 14-3-3σ Q1 and Q4, Gadd45 Q2, Noxa Q1 and Q2, p21-5 Q1 and Q2, p53R2 Q2, Q3 and Q4, puma Q2 and Q4); the representative structure in Fig 2A shows that all four hydrogen bonds are well maintained. The simulations showed that Lys120 also interacted with G or A at positions 1 or 3 in these cases; the only exception is Gadd45 Q1 where Lys120 mainly interacted with G1 (Table 1 and Fig 2B), suggesting that G is preferred for HB; this was not observed in Gadd45 Q3 and p21-5 Q1, suggesting that geometrically the central position is more favorable for Lys120 interactions. (b) When there is a single base mutation, the mutation is at position 1 and the mutated base is C, Lys120 interacted with the central A or G (Noxa Q4, p21-5 Q3 and Q4, Puma Q3) or with both bases at the 2^nd^ and 3^rd^ positions (Gadd45 Q4, Noxa Q3); this is expected since Lys120 is unlikely to interact with G on the other chain at the 1^st^ position. A typical structure is shown in Fig 2C. The interaction with the central base is usually weak if the base is A (Gadd45 Q4, Noxa Q4, p21-5 Q4); however, if T, the interaction is either abolished (p53R2 Q1) or weakened even when G is at the 2^nd^ position (Puma Q3 in Fig 2D); the extra methyl group of T hampered the favorable Lys120 interaction with the 2^nd^ G. (c) If the mutation is at the 2^nd^ position (14-3-3σ Q2), Lys120 interacted with G at the 1^st^ position (Fig 2E); although in this case Lys120 could interact with the A at the 3^rd^ position, the fact that it did not suggests that Lys120 preferred G over A. Reaching the base at the 3^rd^ position is also more difficult due to steric hindrance, requiring the movement of the whole protein. (d) When there were two mutations in a quarter site, Lys120 interacted weakly with the unmutated base (14-3-3σ Q3 and Puma Q1); in the case of 14-3-3σ Q3 the result is expected since both mutated bases were C which does not have HB acceptors; in the case of Puma Q1, the 2^nd^ mutated base was T which was able to form HB; however, there was very little interaction with this base due to the presence of the protruding methyl functional group on T. The only option is the G at the 3^rd^ position, which was also weak for reasons discussed earlier. More dramatic conformational adjustment is needed for better interactions between Lys120 and bases at the 2^nd^ or 3^rd^ positions.

These results indicate that both base position and identity are important for specific binding. Lys120 is able to interact with bases at all three positions, depending on the environment; however, unless more significant conformational adjustment is involved, the binding of Lys120 to bases on the opposite DNA strand is not likely as it was only observed in a quarter site with a small population. The outcome is a unique binding pattern which can lead to a shift of the p53 organization and DNA conformation.

### The stability of Arg280 interaction with base pairs and correlation between Lys120 and Arg280 interactions with DNA

The C at the 4^th^ position is absolutely conserved in all the REs studied here and in most other known p53-REs. The importance of this bp for specificity and affinity has been shown (39,44). In addition, Arg280 formed a salt bridge with Glu281 as part of the HB network in Fig 1b. Arg280 distance fluctuation details are shown in Fig S2 and the HB percentages are summarized in Table 2. Unexpectedly, in many cases the Arg280-C HBs were disrupted for at least two of the four quarter sites for each of the six REs and the salt bridges were also very dynamic (Table 2 and Fig S2), suggesting HB sensitivity to environmental changes, possibly influenced by Lys120-DNA interactions. For example, in the complex of RE **14-3-3σ**, Arg280 HB with DNA was intact for Q1 (Fig 2A) and 4, where Lys120 maintained its HB with the 2^nd^ bp (Tables 2 and 3). This was also the case for **Noxa** Q1 where Lys120-DNA had good interactions at the 2^nd^ and 3^rd^ positions and Arg280 specific interactions were reasonably maintained as well, showing a good correlation between Lys120 and Arg280 interactions. In Q2 of the **14-3-3σ** complex, Lys120 interacted with the base at the 1^st^ position, which loosened the p53 from its original position and reduced the tightness of the Arg280 interaction with the G (Fig 2E, Tables 2 and 3). When Lys120 flipped out of the binding site, as in Q1 of the p53r2 complex, Arg280 also lost both HBs (Fig 2G). Similarly in Noxa Q3, Lys120 interacted with G3, which pushed Arg280 away from its original position, resulting in a conformation in which Arg280 interacted with the DNA backbone (Fig 2C). These results indicate cooperativity between the Arg280 and Lys120 interactions. Interestingly, in the case of Noxa Q4, Lys120 also flipped out of the major groove, yet the Arg280 interactions were still present (Fig 2H). However, such interactions without the concurrent HB of Lys120 nearby are expected to be vulnerable to environmental perturbations. There are also cases where Lys120 interacted with the 2^nd^ base (G or A) but the Arg280 interactions were disrupted. Such changes were observed in the RE **p21**, Q1 and Q2 complexes. In both cases, Arg280 only partially maintained HBs with the bases (Fig 2I).

**Table 2:** Percent salt bridge formation for four salt bridges (A: DNA-R280, B:R280-E281, C: E281-R273, D: R273-DNA).

	Q1				Q2				Q3				Q4			
	A	B	C	D	A	B	C	D	A	B	C	D	A	B	C	D
14-3-3σ	100	0	96	91	0	100	70	0	0	89	99	2	99	20	23	18
GADD45	98	12	68	78	0	54	26	5	0	73	1	0	0	75	48	0
Noxa	0	65	48	75	98	58	51	46	0	77	24	83	92	88	0	5
P21-5	0	80	44	0	45	96	27	33	0	33	29	23	54	57	91	48
P53R2	0	88	34	0	3	77	61	97	90	9	78	12	4	81	0	0
Puma	98	26	95	94	1	3	36	25	2	97	99	13	0	85	54	1

A distance cutoff of 3.5 Å between the donor and acceptor heavy atoms was used in defining the salt bridge.

**Table 3:** DNA bending extent (Degrees) calculated with the program Curves [76], [77] based on 20-bp DNA segment.

Response Element	1^st^ half site	2^nd^ half site
14-3-3σ	18.80	35.25
GADD45	20.03	7.91
Noxa	14.08	16.42
P21	9.94	26.34
P53r2	55.83	12.34
Puma	6.11	8.61

These results indicate that specific HBs of Lys120 and Arg280 not only affect each other, but are also influenced by other interactions, such as the dynamic Arg248 interactions (Fig S3) and the Arg280, Glu281 and Arg273 salt bridge network (Table 2, Fig S4). However, the major factor in determining the conformational changes of the p53-DNA complex is the RE sequence at the Lys120 interaction site, which forces p53 to adjust its conformation locally and consequently the overall organization with respect to the DNA. Interactions at other sites such as those involving Arg280 and Arg248 also adjust their interactions even if the DNA sequences are unchanged. *Thus, even very similar REs, which vary only by a single or a few bps, elicit different patterns of p53-RE interactions perturbing the p53, the DNA and their organization in different ways*.

### The dynamics of the Arg248 interactions

The conformation with Arg248 inserted into the DNA minor groove was captured only in one crystal structure [46]. In others, Arg248 docked only at the edge/surface of the DNA backbone [20], [21], [47]. Arg248 was inside the minor groove at the beginning of our simulations. Once the simulations started, the residue was “ejected” in several complexes and then interacted with the backbone from the outside (Fig S3). As a result, Arg248 shifted away and adopted a conformation similar to those observed in some of the crystal structures. The change in Arg248 interaction patterns would affect the p53 conformation and cause conformational differences among the complexes.

In order to further confirm the relationship between the sequence and the resulting complex conformations, the simulations of 14-3-3σ 1^st^ half site, Gadd45 1^st^ half site, and the Puma 2^nd^ half site were repeated. In 14-3-3σ Q1 (Fig S5A) where Lys120 was expected to interact with the 2^nd^ G base, these HBs were well maintained. In the Gadd45 Q1 (Fig S5B), the respective DNA sequence G1A2A3C4A5 suggests that Lys120 may prefer to interact with the G1 base as observed previously. These interactions were retained reasonably well, with Lys120 positioned within distance capable of HB formation. Because the DNA sequence in Puma Q3 is T1G2A3C4T5, it is expected that the presence of the methyl group on the T base at the 1^st^ position would disrupt the Lys120 HB with the 2^nd^ G base, which was indeed observed (Fig S5C). Comparison of these HB patterns for Lys120 and Arg280 with the corresponding panels in Fig 2A, B and D illustrates consistent and reproducible conformational preferences for a given DNA sequence. The other quarter sites for each of the three complexes were also analyzed and the results were consistent as well.

### Residue fluctuations and allostery

**Figure 3:**
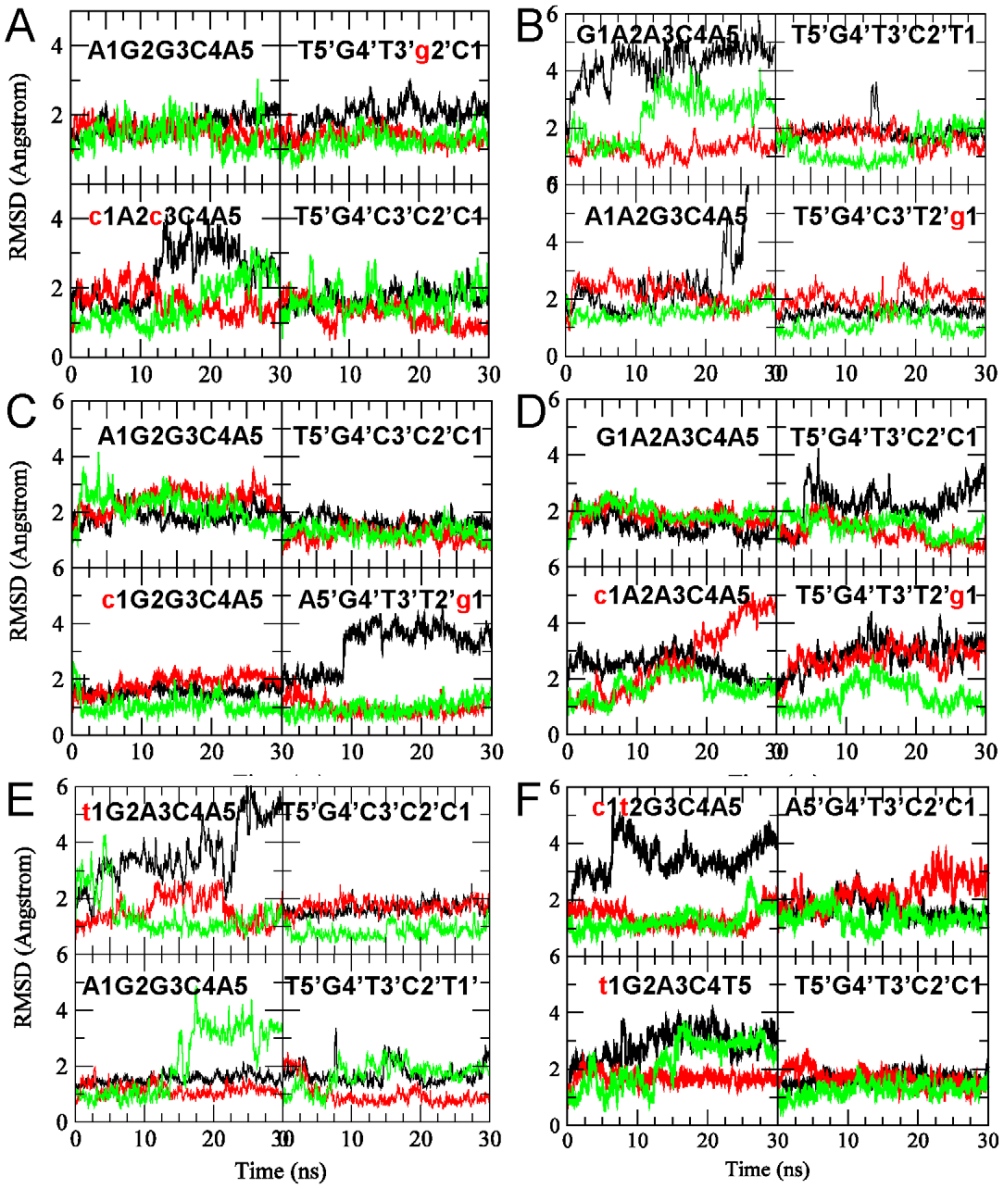
RMS deviations for residues Ly120 (black), Arg280 (red) and Arg248 (green) for each of the p53 core domains. (A)–(F) are for REs 14_3_3σ, Gadd45, Noxa, p21, p53r2, and Puma, respectively. Calculations were performed with the CHARMm RMS module by superimposing the backbone of each p53 monomer onto the initial structure of the respective p53 monomer.

**Figure 4:**
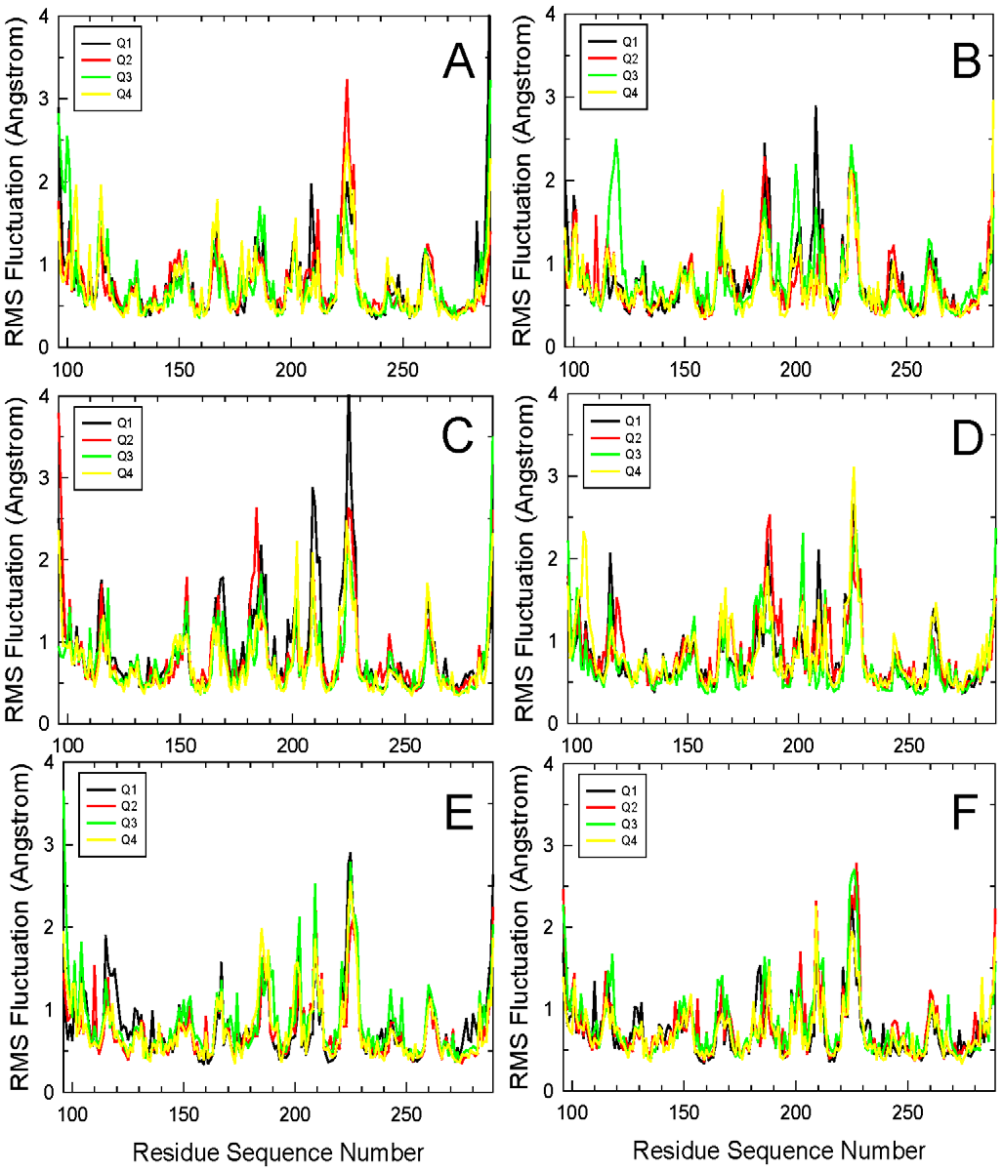
RMS fluctuations for each of the p53 core domain residues. (A)–(F) are for REs 14_3_3σ, Gadd45, Noxa, p21, p53r2, and Puma, respectively. Calculations were performed with the CHARMm RMS module by superimposing the p53 backbones to illustrate the residue deviations from the initial structure. Q1, Q2, Q3 and Q4 stand for quarter sites 1, 2, 3 and 4, respectively for each of the p53-REs. Only the final 5 ns was used in the analysis.

Above, depending on bp identity in each RE the interactions were different. *These subtle differences can allosterically propagate in both DNA and p53*. To characterize these features, conformational changes for both the p53 and DNA were calculated. For p53, the RMS deviation (RMSD) of selected residues and RMS fluctuations (RMSF) of all residues were calculated (Figs 3 and 4). We focused on residues near Lys120 and Arg280. For **14-3-3σ**, large RMSDs were observed for Lys120 in Q3 (Fig 3A); correspondingly, larger RMSF were observed for residues 96–100 and 125–135 next to Lys120 (Fig 4A). For **Gadd45**, Lys120 shifted significantly away in Q3 (Fig 3B), resulting in its large fluctuations and in nearby residues 115–140; although Lys120 in Q1 also had large RMSD, its interactions with the DNA backbone stabilized (Fig 3b). **Noxa** has a large RMSD for Lys120 in Q4 (Fig 3c). However, the RMSF was small, similar to Q1 in Gadd45. In **p21**, Q2 and Q4 had large Lys120 deviations (Fig 3d), slight increase in RMSF nearby in Q2, and large RMSF increase in nearby residues (100–110) in Q4 (Fig 4d). The RMSD for Arg248 were large in Q3 and Q4. Although the RMSF increase for Arg248 was not significant, it was higher for nearby residues 225 and 244. In the case of **p53r2**, large RMSDs of Lys120 in Q1 and of Arg248 in Q3 were observed (Fig 3e); the RMSF of residues 114–136 in the 1^st^ and of residues 230–250 in Q3 also increased correspondingly (Fig 4e). For **Puma**, the RMSD of Lys120 in Q1 and Q3 were relatively large (Fig 3f), resulting in neighboring residues 111 and 125–132 in the 1^st^ and 115–125 in Q3 fluctuating more (Fig 4f). While the RMSD for Arg248 in Q3 was also large, the RMSF of nearby residues changed little, although the pattern of the fluctuation magnitude was somewhat different from the other quarter sites. For the DNA, Table 3 summarizes the bending extent from the last 5 ns of each trajectory, illustrating the allosteric impact on the interactions.

*Thus, adjustments of specific interactions lead to larger fluctuations of nearby residues. In some cases these residues extended to the other side of the protein, suggesting amplified allosteric effect of the DNA on p53, which is likely to be important for selective co-regulator recruitment.*

### Conformational consequences of a change in the interaction patterns

**Figure 5:**
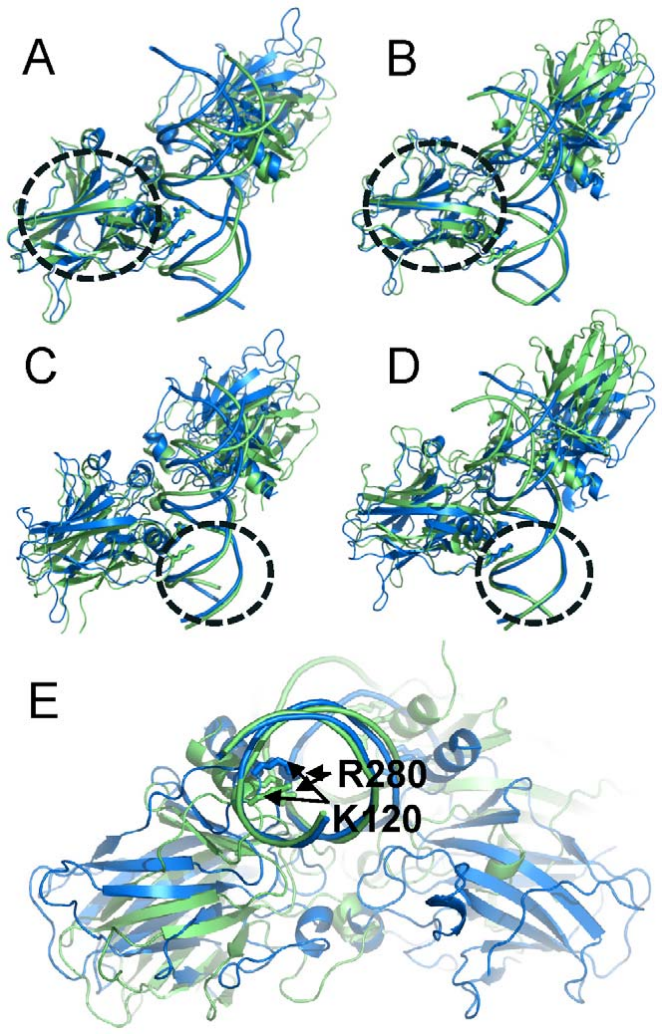
Conformational changes of complex of p53 with the 14-3-3σ 1^st^ half site due to the change in Lys120 interaction pattern. The cartoon representations shown in blue and green are the starting structure and the average structure over the last 5 ns, respectively. In this complex, Lys120 interacted with the 1^st^ G base in Q2, resulting in the shift of the p53 and affecting the organization of the other p53-quarter site interactions. In (A) and (B), the p53 core domain was superimposed for the 1^st^ and 2^nd^ quarter sites, respectively. The superimposition revealed little conformational change in p53. In (C) and (D), the DNA was superimposed for quarter sites 1 and 2, respectively. The superimposition of DNA revealed a large orientation change of p53 with respect to DNA. Structural motifs used for superposition were highlighted with the circle. (E) The structure in a different view of (C) to highlight the shift of residues Lys120 and Arg280 due to the interaction pattern change of Lys120.

**Figure 6:**
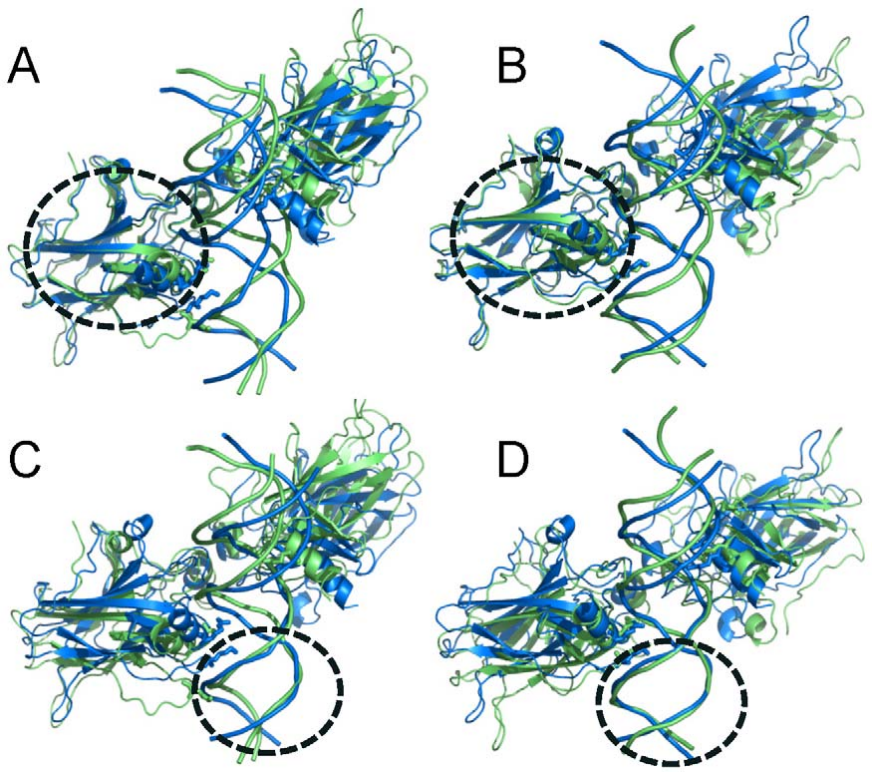
Conformational changes of complex of p53 with the p53r2 first half site due to the change in Lys120 interaction pattern. In Q2 of the complex, Lys120 was pushed out of the major groove and only interacted with the DNA backbone, resulting in the orientation and conformational change of p53. Coloring scheme is the same as in Fig 5. Superimposition schemes are as described in Fig 5 for panels (A), (B), (C) and (D). The superposition of the proteins shows large conformational change of p53 when Lys120 is flipped out in Q1 but the p53 structural deviation is small in Q2 when Lys120 maintains its interactions with the base. The superimposition of the DNA reveals large p53 conformational changes in both quarter sites. Structural motifs used for superposition were highlighted with the circle.

To characterize the conformational changes of the complex elicited by the specific interactions, an angle and a dihedral angle were defined with two atoms from the protein (Cα of S269 and G112) and two from the DNA (C3′ at positions 0 and 4′) (see Fig 1B). These two geometrical parameters were expected to reflect the organizational change of the p53 core domain with respect to the DNA because the two protein atoms are located at the centers of the β-sheet secondary structures and the two DNA atoms belong to the base pairs that are in close contact with the corresponding p53. The calculated results (Table 4) show that the organizations of the p53 monomer-DNA varied to a large extent, ranging from 96 to 112 and from 14 to 44 degrees for the angle and dihedral angle, respectively (Table 4). In the context of the tetrameric p53-DNA complex, such orientation changes for each p53 core domain with respect to the DNA will propagate to the p53 surface away from the DNA binding site. The two examples shown in Figs 5 and 6 illustrate the conformational adjustments between p53 and the DNA. In the 14-3-3σ complex, the RMSDs of both p53 core domains were small (2.5 Å for all atoms) (Figs 5a and 5b). However, when the systems were superimposed with the DNA as the pivot, the p53 orientation changes significantly (Figs 5c and 5d). A major reason for such a change is the interaction pattern. Fig 5e shows that when Lys120 interacts with the G at the 1^st^ position, Lys120, Arg280 and the whole molecule shifted significantly. The significant change of the helix orientation highlights this organizational difference (Fig 5d) which is also reflected in the small dihedral angle (17°) (Table 4). Although no large conformational changes were observed in the p53 itself in this case, allostery can be at play even with minor conformational changes [28]. In the p53 core domain, allosteric fluctuations were observed at locations distant from the allosteric perturbation site [48]. In the case of the p53r2 complex, the flip-out of the Lys120 in one core domain resulted in large protein backbone change (Fig 6a) relative to the other p53 (Fig 6b), leading to a conformational change on the surface of p53 away from the DNA binding site. Both p53 core domains shifted significantly in their orientation with respect to their corresponding DNA quarter sites (Figs 6c, 6d), an outcome of the amplified allosteric effect between the protein and DNA.

**Table 4:** Calculated angle and dihedral angles for the structure averaged over the final 5 ns of the trajectories.

	Angle (degree)				Dihedral (degree)			
	Q1	Q2	Q3	Q4	Q1	Q2	Q3	Q4
14-3-3σ	105	96	105	107	31	17	27	28
gadd45	110	105	101	104	28	34	30	27
noxa	100	99	102	105	15	23	28	30
p21	103	108	101	104	23	23	30	22
p53r2	98	107	104	99	19	32	32	14
puma	103	101	112	103	44	16	24	25

Q1, Q2, Q3 and Q4 stand for the four quarter sites. The angle and the dihedral were defined in fig 1b.

### Correlation between the Ly120 and Arg280 movement

**Figure 7:**
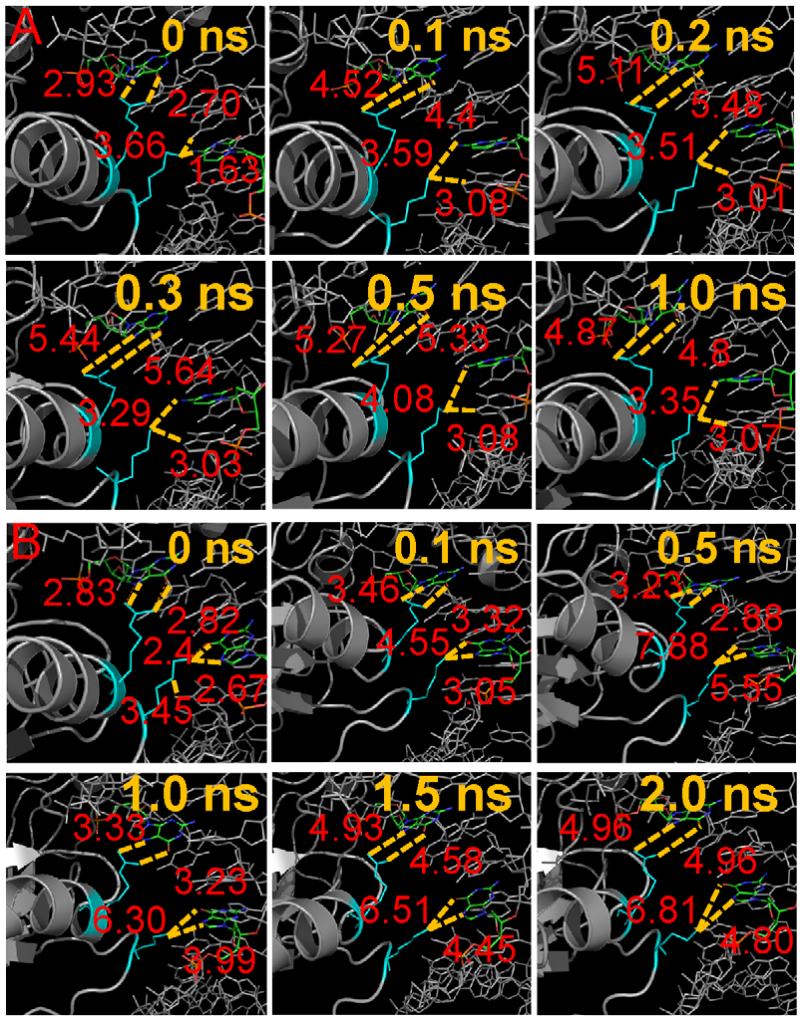
Selected sequences of events for correlated movements of residues Ly120 and Arg280. (A) and (B) snapshots of conformations from the trajectory of 14-3-3σ quarter site 2 and those of p53R2 quarter site 1, respectively. Color coding of the residues are the same as in Fig 2. In 14-3-3σ quarter site 2 complex (DNA sequence is T5′G4′T3′G2′C1′), Lys120 preferred to make hydrogen bond with the G base at the 1′^st^ position in the complementary chain and have to move its side chain. In the p53R2 quarter site 1 complex (DNA sequence is T1G2A3C4A5), the presence of Methyl group of T base at the 1^st^ position destabilized the Lys120 interactions with the G base at the 2^nd^ position, leading to the pull-away of Lys120 from the major groove to avoid the steric clash with the Methyl group. Hydrogen bond distances were highlighted with dotted yellow lines.

Lys120 and Arg280 are the two major factors that determine the binding specificity to the p53-REs. While Arg280 mostly interacts with the G base at the 4^th^ position within a quarter site, the adjustment of Lys120 interaction may affect the Arg280 interaction since these two residues are next to each other. To see if the two interactions are correlated, covariance map (Fig S6), interaction energy between the two residues (Fig S7), and the correlation between the HB distances of the two residues with DNA bases (Fig 7) were calculated. The covariance map revealed that the movements of residues 115–125 were negatively correlated with different portions of the p53 core domain, depending on the DNA sequence. One common negatively correlated portion was residues from 175–185, suggesting that the movement of the residues near Lys120 will affect the residues at the dimerization interface. Since these correlations were quarter-site specific, it is difficult to draw a general rule regarding the correlation between the conformational change and the RE type.

The interaction energies between the two residues showed near zero net interaction energy (e.g. 14-3-3σ Q1, Q2, Q4) when Lys120 and Arg280 assumed near crystal structure conformation. When Lys120 popped out of the binding pocket, the interaction energies became either more favorable (14-3-3 σ Q3, Noxa Q4, Puma Q1) (Fig S7A, C, F), or less favorable (Gadd45 Q1, p21-5 Q2, Q4), or mostly changed little when Lys120 did not flip out. These results suggest that the altered packing of Lys120 triggers the readjustment of the Arg280 interactions with the new environment. Such a relationship is also reflected in the HB distances. Fig S8 shows that when the Lys120 HB broke, those of Arg280 also quickly disrupted (14-3-3σ Q2, Q3; Gadd45 Q3, Q4; p53R2 Q1; Puma Q3). Although in some cases the Lys120 HB disruption did not necessarily result in the disappearance of Arg280 HBs within the limited simulation time (Noxa Q4; p21-5 Q4; Puma Q1), their stability in the long run is likely to be compromised due to the lack of tight packing.

To further demonstrate the correlation between the movement of Lys120 and Arg280, we present snapshots from two trajectories. Fig 7 shows that the conformational changes happened very early in the trajectories. For 14-3-3σ Q2 (Fig 7A), the distance between Lys120 and the C base at the 2^nd^ position of the quarter site was too close (1.63 Å) and too far (3.66 Å) to interact with the G base at the same position on the complementary chain in the initial structure. After 0.01 ns, Lys120 shifted away from the 2^nd^ bp moving toward the 1^st^ bp, causing the weakening of the neighboring Arg280 HB (Fig 7A) with subsequent adjustment of the interactions of both residues with the DNA. While Lys120 was settling with the G1 base from 0.01 to 1 ns, Arg280 continued to lose contact with G4 base, shown by the longer interaction distances. In the p53R2 Q1 trajectory, both Lys120 and Arg280 HBs were nicely organized in the starting structure (0 ns) (Fig 7B). Because of the protruding methyl group of the T base at the 1^st^ position of the quarter site, Lys pulled away from the G base at the 2^nd^ position to avoid steric clash (0.1 ns) and drifted further away from the starting point (0.5 ns). While Lys120 was searching for favorable positions after pulling away from the major groove, Arg280 started to fray and the HB distance from the G base became longer and out of range from 1 to 1.5 ns. The final settled conformation is similar to that at 2 ns (Fig 7B). When compared with structures where both Lys120 and Arg280 maintained their HBs with the 2^nd^ and 4^th^ bases, these two examples clearly demonstrate that the movement of Arg280 or the loss of Arg280 HBs was the outcome of the Lys120 movement.

## Discussion

In each quarter site, the p53-REs largely conform to the consensus sequence and are highly similar to each other. This raises a key question that has been largely overlooked [12], [13]: how does the small, often minor sequence variation of a single or few bps, translate into vastly different functional consequences, spelling transcription activation or repression? The *in vitro*, or cell-based affinity experiments do not necessarily correlate with the functional consequences [8], [9] and the sparseness of available experimental structures makes such an investigation highly challenging [49]. Our computational results provide insight into this crucial question, illustrating how minor DNA sequence changes can impact subsequent recognition events which in turn determine the *functional* outcome. We show that subtle conformational changes elicited by DNA sequences which can differ by as little as a single bp can result in altered p53 core domain organization and protein surface dynamics. The DNA is an allosteric effector; slightly different RE sequences lead to minor alterations in the core domain-DNA interactions. The core domain conformational changes may propagate and thus allosterically impact the full protein including the N- and C-terminal domains, providing preferred surfaces for recruitment of specific co-regulators such as STAGA [50], [51], CBP/p300 and HDM2 [52]. The amplified allosteric changes at the p53 surface can select different co-regulators [13]. Conformational selection and population shift have been proposed to play a key role in biomolecular recognition [26]–[28], [53], [54]. Cofactor binding can also affect RE selectivity by transcription factors through an alternative allosteric mechanism [12], [13]. In this case, the prior binding of the co-regulator will shift the population of the transcription factor leading to altered DNA-binding site conformation. ASPPs (apoptosis-stimulating proteins of p53) for example, when bound to p53 core domain, can shift the p53 ensemble enhancing a conformation that favors binding to specific p53-REs [12], [13], [55]. In light of the findings from this work, it is likely that the ASPP binding changes the loop L1 conformation of the p53 core domain, which has been demonstrated to be of crucial importance to the specificity of RE binding. The structured L1 loop could govern the allosteric pathway mediating these binding sites.

The features captured here are only part of the story. DNA sequence variation can also code for the differential binding of p53 family proteins. For example, RE2 of the target gene GDF15 contains sequence variations that allow only p53 but not p63 and p73 binding [56]. This may explain why DNA sequences GGG, GGA or AGG all have similar binding patterns and affinities with p53 [20] but in combination can exclude the binding of other proteins. We further note that although our results clearly show that the p53-DNA interaction patterns and conformational and residue fluctuations vary with DNA sequence, allostery may not be saliently evident in some cases. The allosteric structural perturbations observed in experiments or simulations are the sum of multiple, major and minor pathways [57] and these may not be detected in the current analysis. The transmission of the signal over long distances may be difficult to observe in short MD simulations, and conformations that are relevant for cofactor binding may have high barriers to go through or higher energy, i.e. be less populated [58] and difficult to observe in simulations [59] and in experiment [58], [60]. However, recently a series of crystal structures coupled with biochemical and cell-based assays have shown how the glucocorticoid (GR) REs that vary by even a single bp can lead to different GR conformations at a cofactor binding site, thus affecting GR regulatory activity [13], [14], [40].

The cellular network, which reflects the environment, contributes critically to transactivation selectivity [12], [13] and p53 acetylation was shown to be related to the differential activation of apoptosis or cell cycle arrest [61], [62]. Methylation of cofactors such as the heterogeneous nuclear ribonucleoproteins hnRNP K can hamper the recruitment of p53 to the REs [63]. Similarly, arginine methylation in p53 may also control target gene selectivity [64]. Post-translational modifications of p53, including phosphorylation and acetylation [65], allosterically alter its activity. Covalent modifications provide an added level of cellular network regulation, in addition to protein co-regulator availability which is also regulated by the network in response to changes in the cellular environment.

Although not addressed here, sequences flanking the REs are important for the overall organization of the complex, likely also via allosteric effects, combinatorial assembly of other transcription factors binding in these regions [13] and chromatin remodeling. Flanking segments assist in co-regulator transcription recruitment, as shown for the human BAX promoter [66] which can allosterically trigger conformational changes in p53 and neighboring DNA sequences, rendering the binding surface that is specific for cofactor binding. Further, the p53 core domain dimers interactions with DNA and with each other are primary factors responsible for specific cooperative DNA binding, with the interactions enhanced in the full-length protein [16]. The C-terminal domain is also involved in the interactions. While not included here, allosteric effects observed in this work further implicate the conformations of other p53 domains.

p53-REs can have spacers with sizes ranging between 1–20 bps. p53-REs with 5- or 6-bp insertions have the weakest binding even with full fledged p53 [67]. p53 dimer-dimer cooperative interactions are important for function [17], and such cooperative interactions are unlikely for systems with 3–6 (and probably more) bp spacers [17]. In some cases, there is only one RE half site and there can still be significant transcriptional activity [68]. In these cases, the allosterically amplified p53 conformational changes induced by half-site DNA could still be large enough for specific recruitment of transcription co-regulators, while the second p53 dimer may bind DNA non-specifically. The notion that even when there is one bp change allosteric effects can still specify biomolecular recognition and hence determine function supports the likelihood that specificity of the 10-bp half site p53-REs is sufficient.

Selective p53-related gene expression requires p53 binding to DNA and pre- and post-DNA binding regulatory events such as modifications of both p53 protein and DNA [69], the recruitment of transcriptional cofactors and RE availability. In a recent example [70], there exists an identical transcriptional target in apoptosis promoters such as BAX and Puma that was selectively blocked by SMAR1 expressed under mild DNA damage conditions. Under severe DNA damage, other factors displace the SMAR1 protein to allow the initiation of apoptotic processes. The actual repression of the relevant genes might involve direct p53 binding onto the target sites [71]. While selective transcription mechanisms are still unclear [12]–[14], our findings here on the p53-RE binding-induced selectivity and future developments are expected to provide further insight into the mechanisms of RE selectivity and the regulation of the first step in transcription initiation.

To conclude, here we describe a molecular dynamics study of the p53-DNA interaction, particularly focusing on amino acids that make direct contact with DNA bases. We found that the side chain of Lys120 was able to make a number of alternative contacts with DNA bases at positions 1–3. This observation is consistent with low experimentally observed sequence specificity for p53 binding. We further observed that the conserved interaction of Arg280 with its cognate base pair may be broken in some cases, and that Arg248 is more likely to interact with the DNA backbone than make specific contact with DNA. We show that variant Lys120 interactions with bases at different positions can shift the overall p53-DNA interaction patterns, and *how* the conformation adopted by Lys120 influences the conformation adopted by other DNA-interacting residues. Most interestingly, the relative orientation of the p53 core domain and DNA changes depending on the sequence of the response element. This leads us to conclude that different response elements will result in different organization of p53-DNA complexes, potentially exposing different surfaces. This, in turn, could result in recruitment of different co-factors and explain the different functionality of response elements whose sequence differs by only a few nucleotides.

## Methods

### MD simulation protocol

MD simulations were performed on 12 p53 dimer-DNA half site complexes constructed based on the p53-DNA crystal structure with the PDB code 1tsr [46]. The detail construction methods of the models were described in the next section. Each system was solvated with a rectangular TIP3P water box [72] with a margin of at least 10 Å from any edge of the box to any protein or DNA atom. Solvent molecules within 1.6 Å of the DNA or within 2.5 Å of the protein were removed. The systems were then neutralized by adding sodium ions. The resulting systems were energy minimized for 1000 steps before the dynamic run using the CHARMm program [73] and the CHARMm 22 and 27 force field for the protein and nucleic acid, respectively [74]. The production MD simulations were performed at temperatures of 300 degrees Kelvin using the NAMD program [75] and the CHARMm force field. Periodic boundary conditions were applied and the non-bonded lists were updated every 20 steps. The NPT ensemble was applied and the pressure kept at 1 atom using Langevin-Nose-Hoover coupling. SHAKE constraints on all hydrogen atoms and a time step of 2 fs and a nonbonded cutoff of 12 Å were used in the trajectory production. The sizes of the systems were about 110,000 atoms and the duration for each simulation was 30 ns.

### Modeling of p53 dimer-DNA complexes for each p53-RE half site

The p53 core domain dimer-half site DNA complex was generated based on the crystal structure template (PDB code: 1tsr) [46], as described earlier [44], [45]. Briefly, we used two copies of the p53 monomer-DNA complex crystal structure and then superimposed the 10 consensus base pairs from the two copies of the extracted p53-DNA complex in reverse order so that the two copies of p53 were bound to two consecutive quarter sites of the DNA. The resulting p53 dimer-DNA complex structure ensures specific DNA-p53 binding and that the two copies of p53 have a C_2_ symmetry, with formation of the two salt bridges between Arg180 and Glu181 from the H1 helices of the p53 core domains. The DNA sequences that capped the 5′ and 3′ ends were 5′-ATAATT-3′ and 5′-ATTAA-3′, respectively. Each base pair that was different from the target sequence was mutated by removing the atoms in the base motif and these atoms were regenerated with GENERATE module in the CHARMm program. The systems were then minimized for 2000 steps with SD algorithm, the mutated base pairs were allowed to move with the NOE restrictions that all the distances between hydrogen bond partners (heavy atoms) were within 2.6 and 3.0 Å. The rest of the system was not allowed to move by applying a force constant of 2 kcal/mol/å during the minimization. The obtained structures were then further minimized for 1000 steps with the ABNR algorithm without any restriction. The models obtained in such a manner yielded reasonable local and overall conformations and served as the starting structure for the MD simulations. For the three duplicate simulations for the purpose to ensure the reliability of the results, additional 1000 steps with the ABNR algorithm was applied before the start of MD trajectories.

## Supporting Information

Figure S1 Hydrogen bond distances between Lys120 of p53 and the base pairs at positions 1–3 of the p53-RE quarter site. (A)–(F) are for REs 14-3-3σ, Gadd45, Noxa, p21, p53r2, and Puma, respectively. 6 distances are shown for each of the four quarter sites, with 1a and 1b from the 1^st^, 2a and 2b the 2^nd^, and 3a and 3b the 3^rd^ position base pairs. If the base pair is a GC or CG, the two distances between Lys120 and the base pair are for O6 and N7. If the base pair is an AT or TA then the two distances are for atoms O4 and N7 shown in Figure 1.
(1.80 MB TIF)

Figure S2 Hydrogen bond distances between Arg280 of p53 and base pairs at position 4 of the p53-RE quarter site and between Arg280 and Glu281. (A)–(F) are for REs 14_3_3σ, Gadd45, Noxa, p21, p53r2, and Puma, respectively. 6 distances were shown for each of the four quarter sites. 1a and 1b are for distances between Arg280 and the base pair. 2a, 2b, 3a and 3b are the distances between Arg280 and Glu281.
(1.43 MB TIF)

Figure S3 Interaction distances between Arg248 of p53 and DNA backbone at positions 4–5 of a p53-RE quarter site. (A)–(F) are for REs 14_3_3σ, Gadd45, Noxa, p21, p53r2, and Puma, respectively. Two distances were shown for each of the four quarter sites.
(1.51 MB TIF)

Figure S4 Interaction distances between Arg273 of p53 and DNA backbone and between Arg273 and Glu281. (A)–(F) are for REs 14_3_3σ, Gadd45, Noxa, p21, p53r2, and Puma, respectively. Two distances were shown for each of the four quarter sites.
(0.55 MB TIF)

Figure S5 Average structures of the p53-DNA complex over the last 5 ns of the Lys120 and Arg280 binding sites for three duplicate simulations. (A) 14-3-3σ 1^st^ half site Q1. (B) Gadd45 1^st^ half site Q1. (C) Puma 2^nd^ half site Q3. Lys120 and Arg280 are colored in cyan and the 2^nd^ and 4^th^ bases are colored based on atom type. Hydrogen bonds formed between Lys120 and the 2^nd^ base or between Arg280 and the 4^th^ base are shown in dotted yellow lines. The calculations were performed with the CHARMm analysis module COOR DYNAMICS.
(3.83 MB TIF)

Figure S6 Calculated covariance map of Cα atoms with each of the p53 core domain. Red and purple denote positive and negative correlations, respectively. (A)–(F) are for REs 14-3-3σ, Gadd45, Noxa, p21, p53r2, and Puma, respectively. For clarity and to show the impact of motions of residues near Lys120, only residues 100–140 were plotted in the Y axis.
(3.31 MB TIF)

Figure S7 Calculated Lys120-Arg280 interaction energies for each p53 core domain. (A)–(F) are for REs 14-3-3σ, Gadd45, Noxa, p21, p53r2, and Puma, respectively. For clarity and to show the impact of motions of residues near Lys120, only residues 100–140 were plotted in the Y axis.
(1.11 MB TIF)

Figure S8 Lys120-Arg280 hydrogen bond distances for each p53 core domain. (A)–(F) are for REs 14-3-3σ, Gadd45, Noxa, p21, p53r2, and Puma, respectively. For simplicity, only one distance for each Lys120 and Arg280 was plotted. Lys120 hydrogen bond distance was based on the average of the NZ (Lys120)-O6 (G2) and NZ-N7 (G2) distances, and Arg280 distance the average of NH1 (Lys120)-O6 (G4′) and NH2 (Lys120)-N7 (G4′).
(1.72 MB TIF)
