# Supplementary figures and images for: Lysine120 Interactions with p53 Response Elements can Allosterically Direct p53 Organization
Source: PLoS Comput Biol. 2010 Aug 5;6(8):e1000878. doi: 10.1371/journal.pcbi.1000878 (PMC2916859; doi:10.1371/journal.pcbi.1000878)

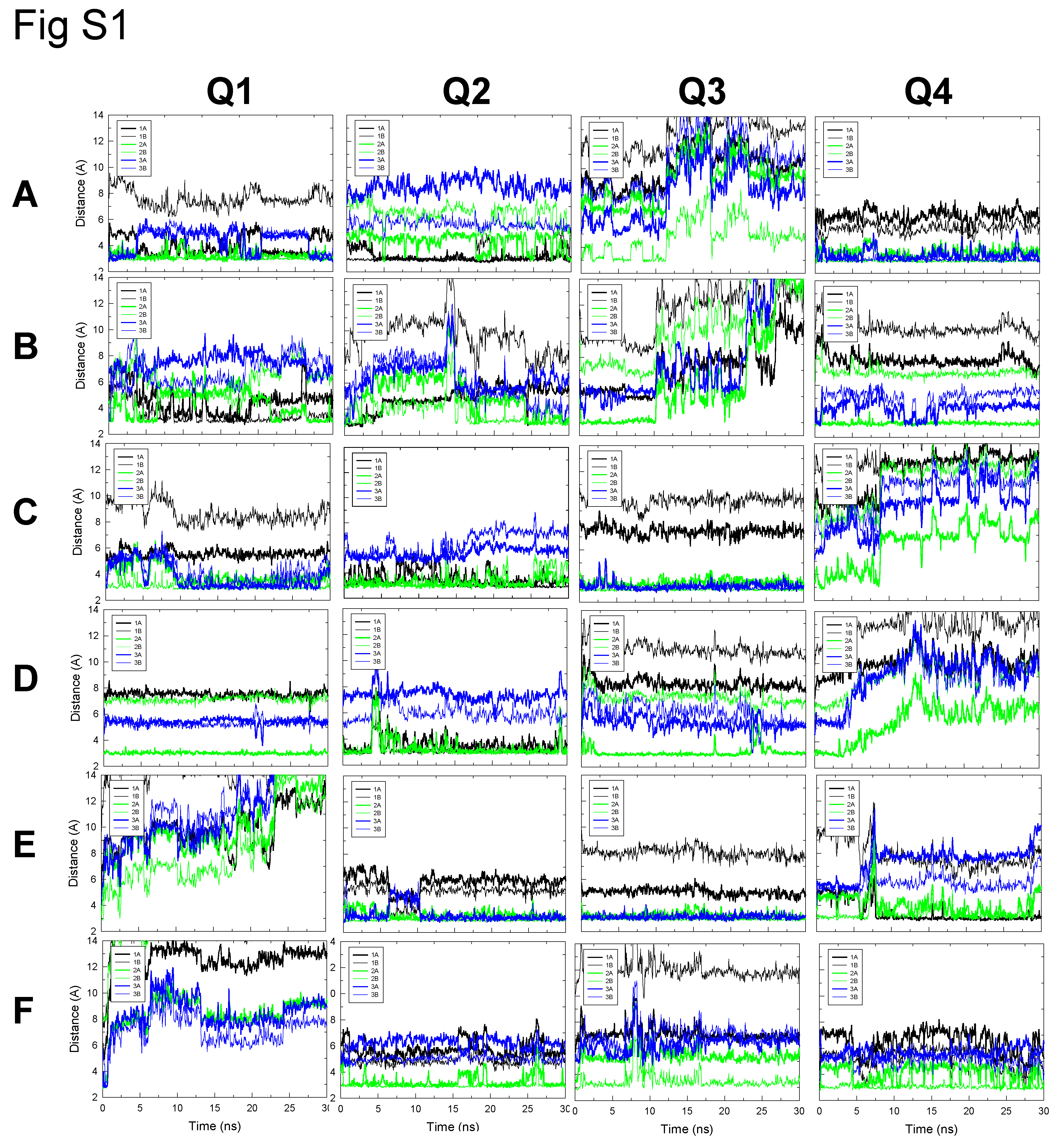

Supplement: Figure S1 — Hydrogen bond distances between Lys120 of p53 and the base pairs at positions 1–3 of the p53-RE quarter site. (A)–(F) are for REs 14-3-3σ, Gadd45, Noxa, p21, p53r2, and Puma, respectively. 6 distances are shown for each of the four quarter sites, with 1a and 1b from the 1st, 2a and 2b the 2nd, and 3a and 3b the 3rd position base pairs. If the base pair is a GC or CG, the two distances between Lys120 and the base pair are for O6 and N7. If the base pair is an AT or TA then the two distances are for atoms O4 and N7 shown in Figure 1. (1.80 MB TIF) [file pcbi.1000878.s001.tif]

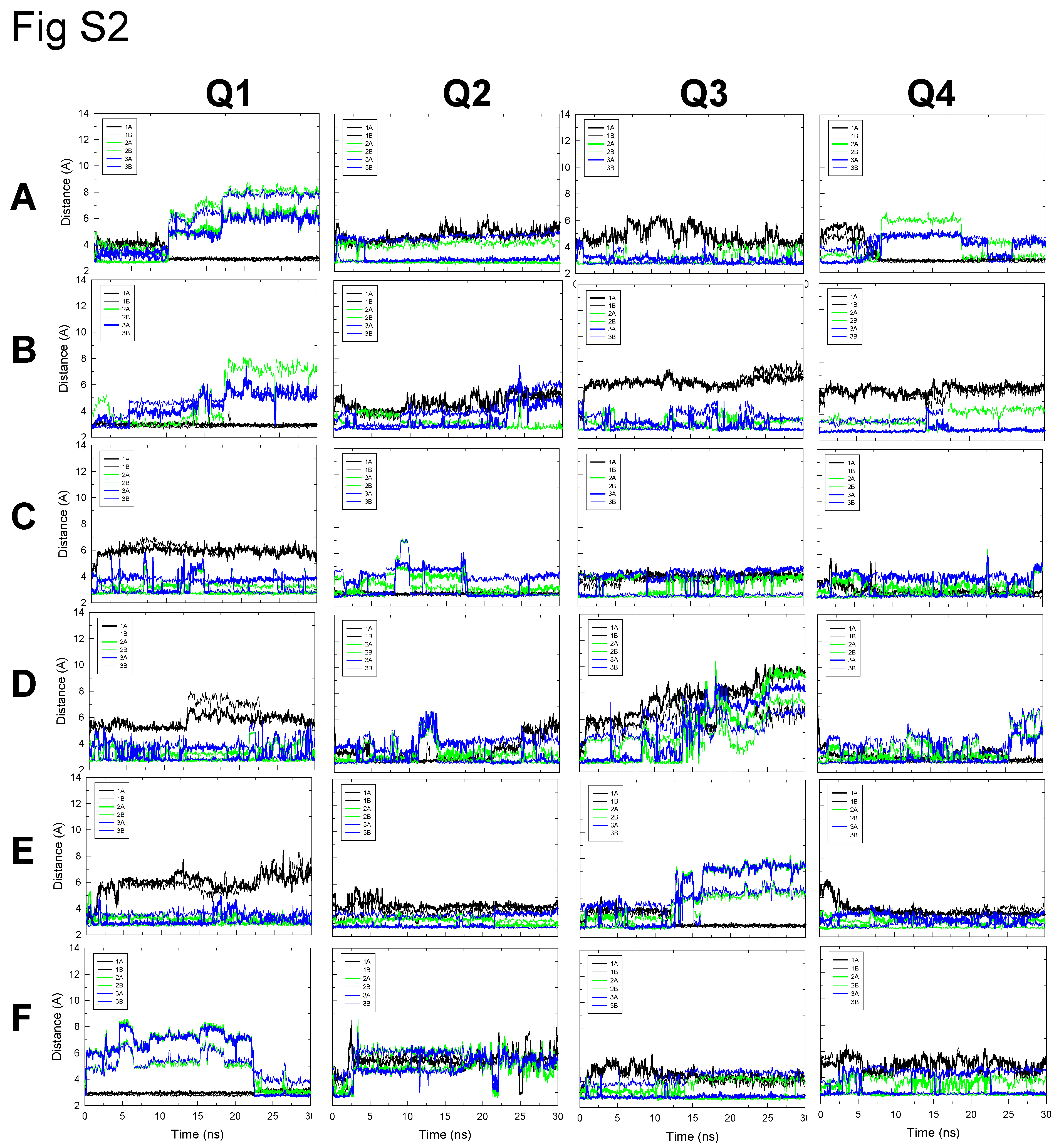

Supplement: Figure S2 — Hydrogen bond distances between Arg280 of p53 and base pairs at position 4 of the p53-RE quarter site and between Arg280 and Glu281. (A)–(F) are for REs 14_3_3σ, Gadd45, Noxa, p21, p53r2, and Puma, respectively. 6 distances were shown for each of the four quarter sites. 1a and 1b are for distances between Arg280 and the base pair. 2a, 2b, 3a and 3b are the distances between Arg280 and Glu281. (1.43 MB TIF) [file pcbi.1000878.s002.tif]

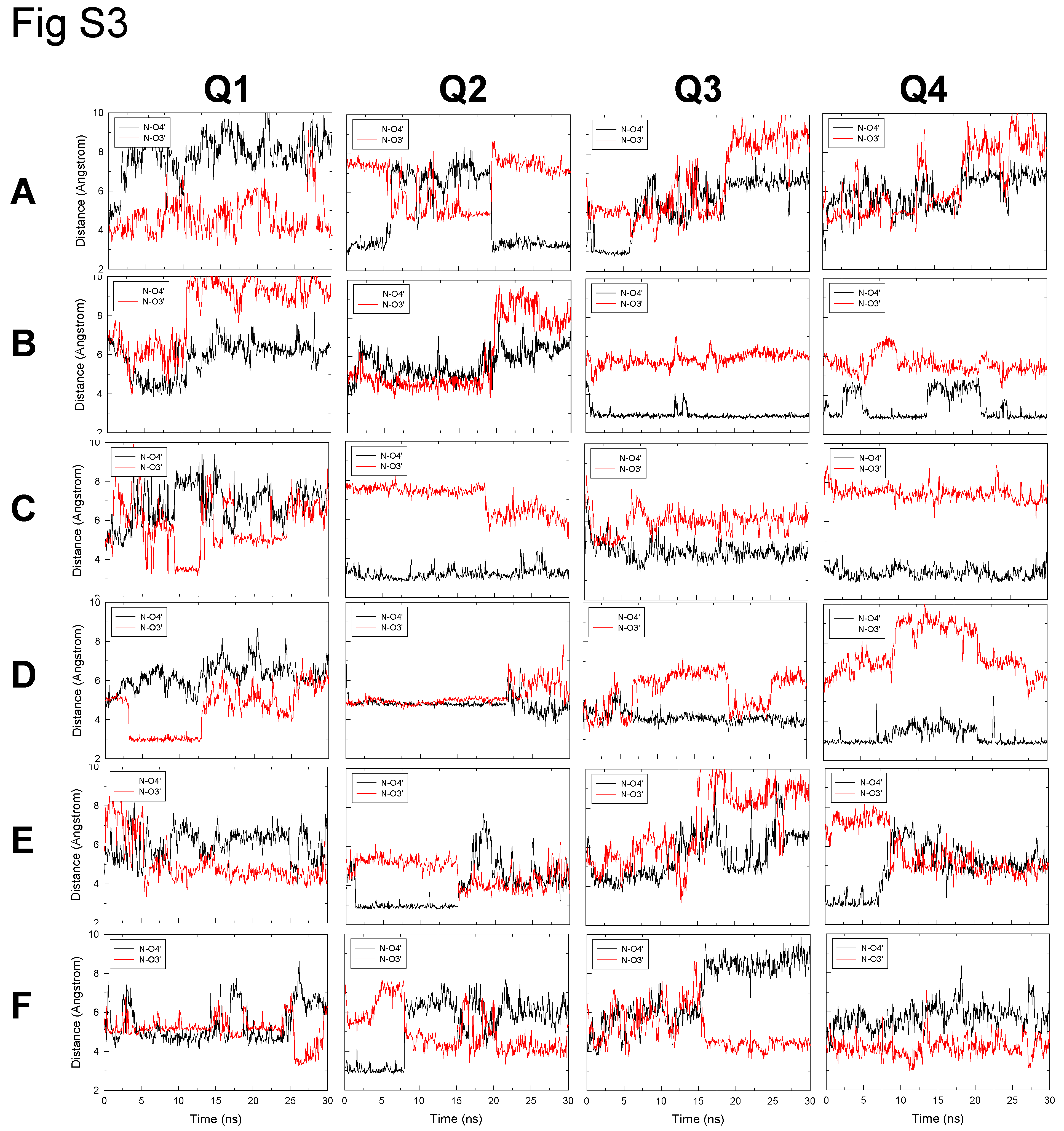

Supplement: Figure S3 — Interaction distances between Arg248 of p53 and DNA backbone at positions 4–5 of a p53-RE quarter site. (A)–(F) are for REs 14_3_3σ, Gadd45, Noxa, p21, p53r2, and Puma, respectively. Two distances were shown for each of the four quarter sites. (1.51 MB TIF) [file pcbi.1000878.s003.tif]

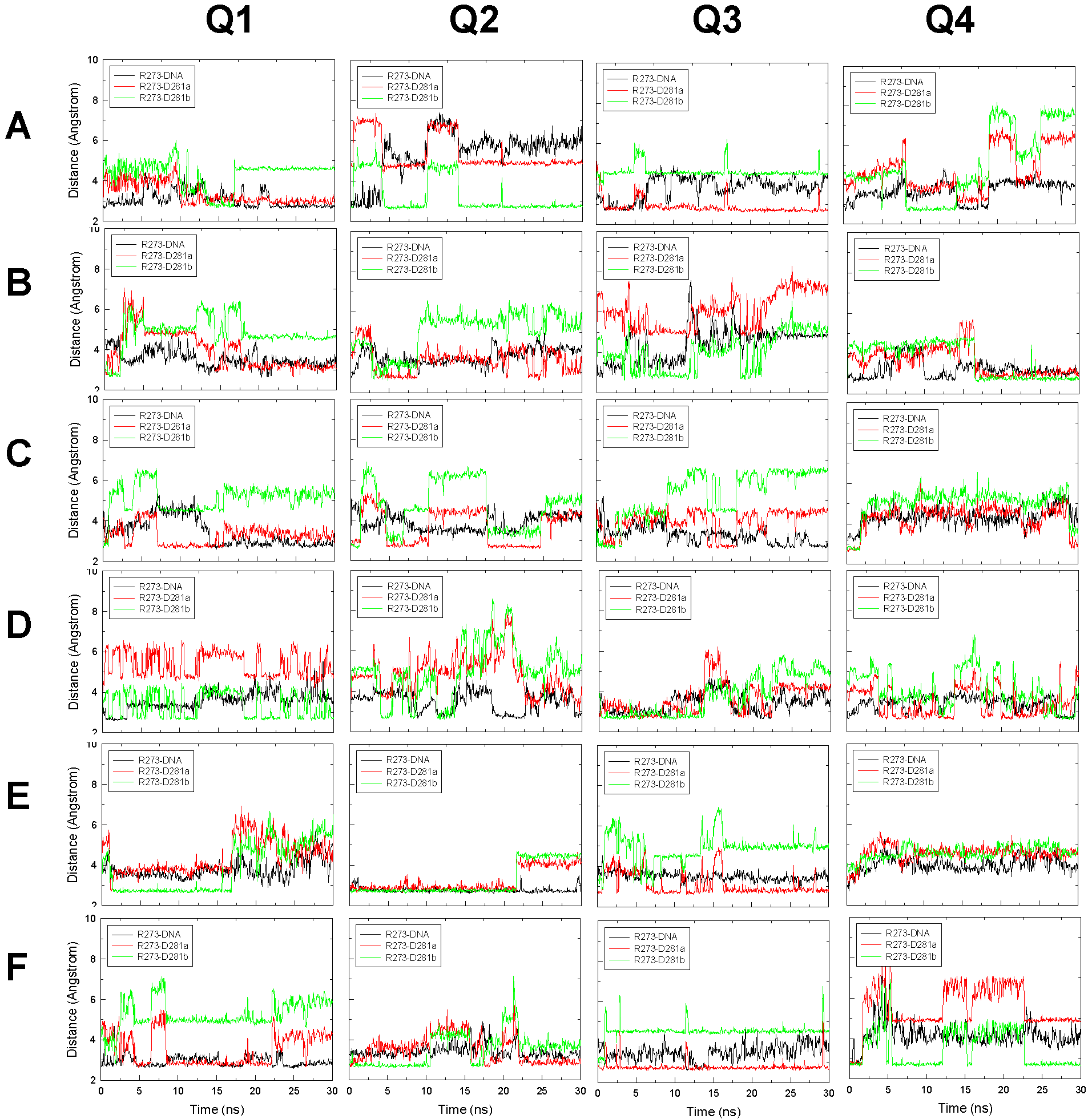

Supplement: Figure S4 — Interaction distances between Arg273 of p53 and DNA backbone and between Arg273 and Glu281. (A)–(F) are for REs 14_3_3σ, Gadd45, Noxa, p21, p53r2, and Puma, respectively. Two distances were shown for each of the four quarter sites. (0.55 MB TIF) [file pcbi.1000878.s004.tif]

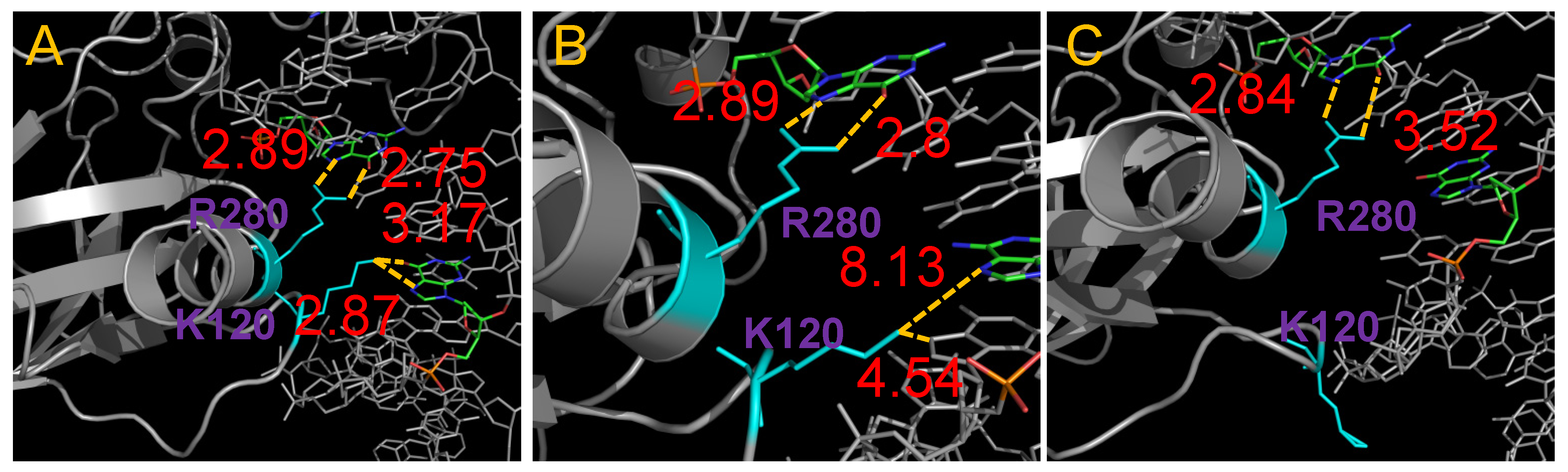

Supplement: Figure S5 — Average structures of the p53-DNA complex over the last 5 ns of the Lys120 and Arg280 binding sites for three duplicate simulations. (A) 14-3-3σ 1st half site Q1. (B) Gadd45 1st half site Q1. (C) Puma 2nd half site Q3. Lys120 and Arg280 are colored in cyan and the 2nd and 4th bases are colored based on atom type. Hydrogen bonds formed between Lys120 and the 2nd base or between Arg280 and the 4th base are shown in dotted yellow lines. The calculations were performed with the CHARMm analysis module COOR DYNAMICS. (3.83 MB TIF) [file pcbi.1000878.s005.tif]

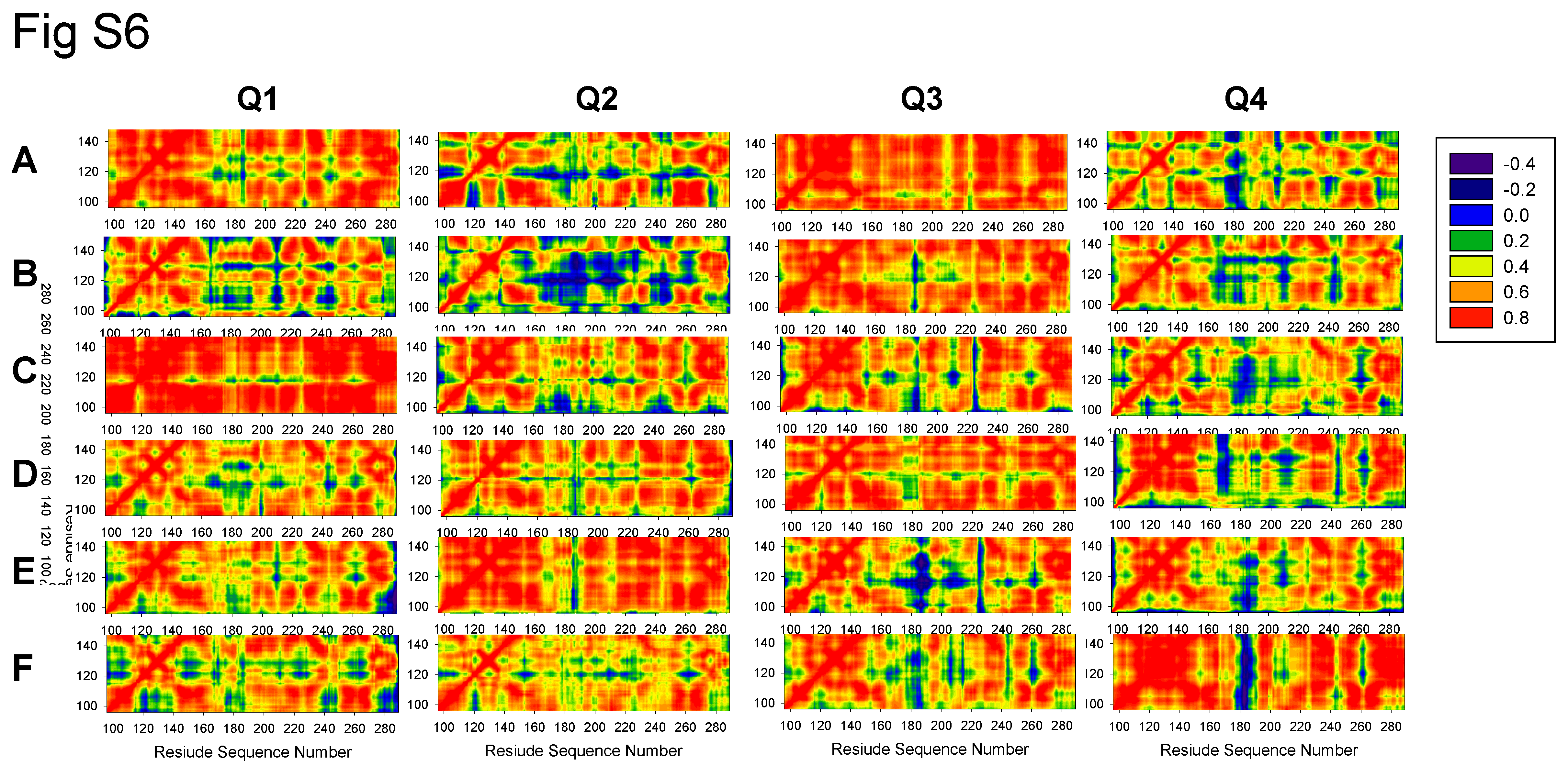

Supplement: Figure S6 — Calculated covariance map of Cα atoms with each of the p53 core domain. Red and purple denote positive and negative correlations, respectively. (A)–(F) are for REs 14-3-3σ, Gadd45, Noxa, p21, p53r2, and Puma, respectively. For clarity and to show the impact of motions of residues near Lys120, only residues 100–140 were plotted in the Y axis. (3.31 MB TIF) [file pcbi.1000878.s006.tif]

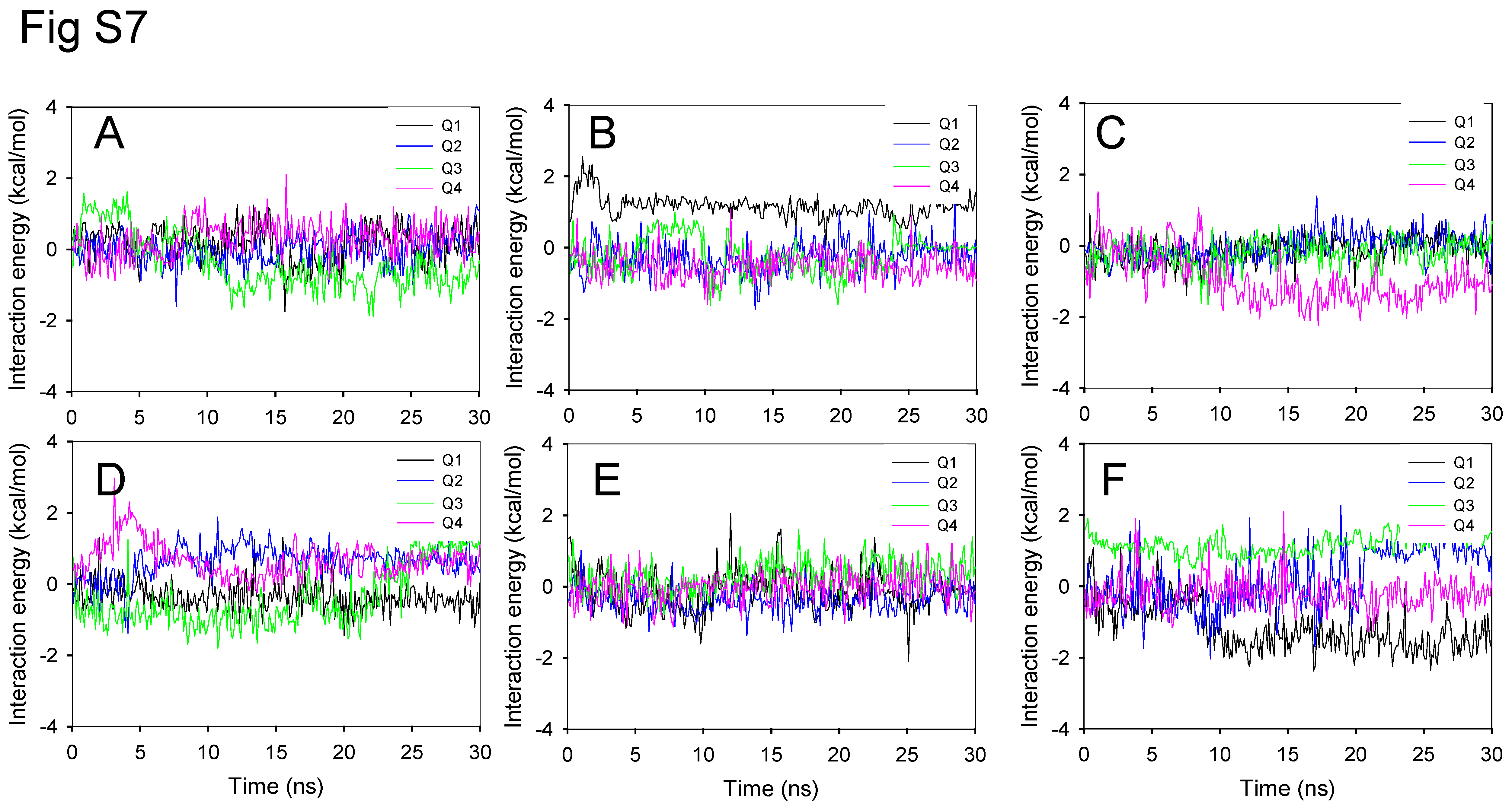

Supplement: Figure S7 — Calculated Lys120-Arg280 interaction energies for each p53 core domain. (A)–(F) are for REs 14-3-3σ, Gadd45, Noxa, p21, p53r2, and Puma, respectively. For clarity and to show the impact of motions of residues near Lys120, only residues 100–140 were plotted in the Y axis. (1.11 MB TIF) [file pcbi.1000878.s007.tif]

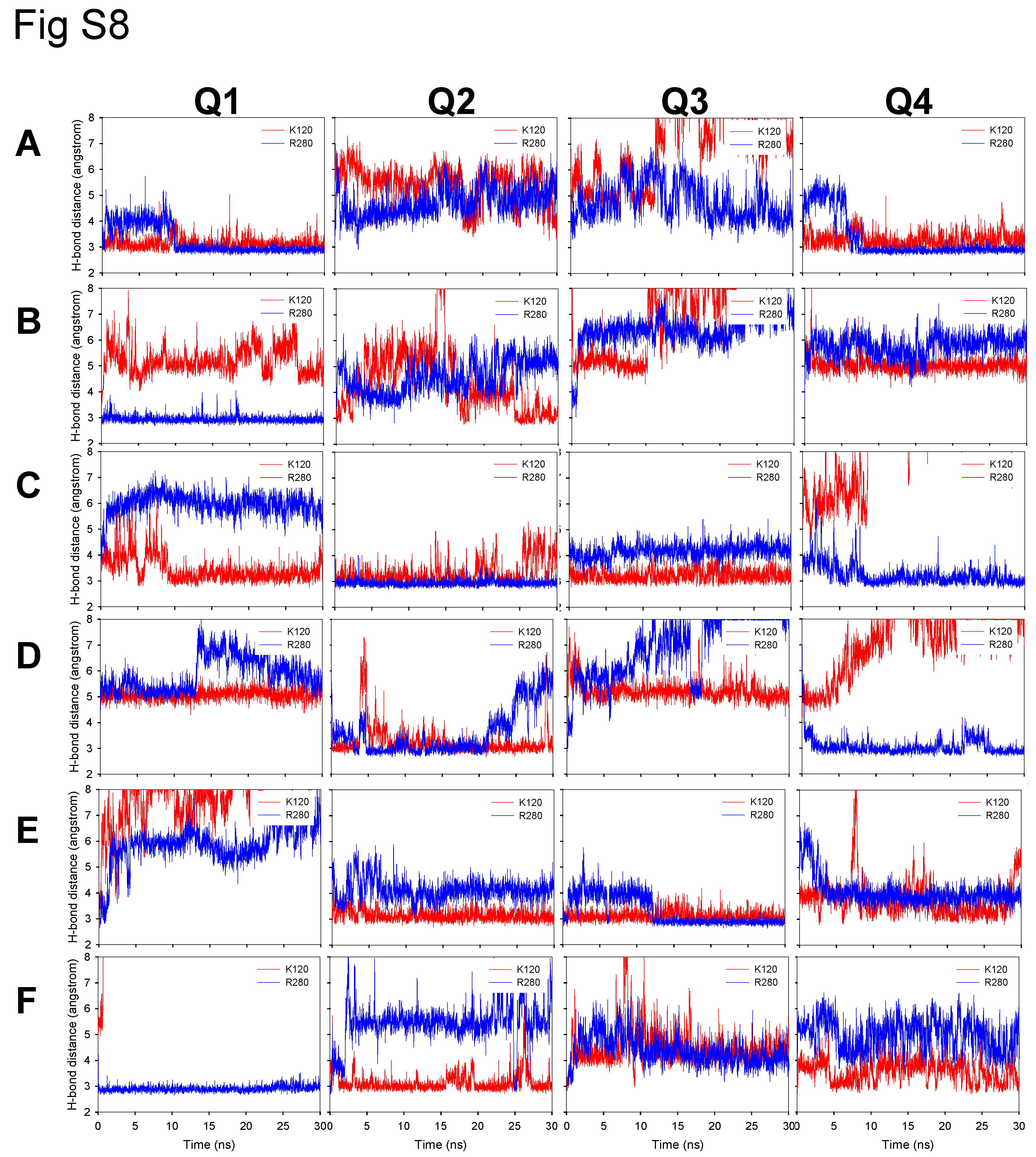

Supplement: Figure S8 — Lys120-Arg280 hydrogen bond distances for each p53 core domain. (A)–(F) are for REs 14-3-3σ, Gadd45, Noxa, p21, p53r2, and Puma, respectively. For simplicity, only one distance for each Lys120 and Arg280 was plotted. Lys120 hydrogen bond distance was based on the average of the NZ (Lys120)-O6 (G2) and NZ-N7 (G2) distances, and Arg280 distance the average of NH1 (Lys120)-O6 (G4′) and NH2 (Lys120)-N7 (G4′). (1.72 MB TIF) [file pcbi.1000878.s008.tif]
